# Effect of COVID-19 on Lungs: Focusing on Prospective Malignant Phenotypes

**DOI:** 10.3390/cancers12123822

**Published:** 2020-12-18

**Authors:** Pritam Sadhukhan, M. Talha Ugurlu, Mohammad O. Hoque

**Affiliations:** 1 Department of Otolaryngology-Head and Neck Surgery, Johns Hopkins University School of Medicine, Baltimore, MD 21231, USA; psadhuk1@jhmi.edu (P.S.); mugurlu1@jhmi.edu (M.T.U.); 2 Department of Oncology, Johns Hopkins University School of Medicine, Baltimore, MD 21231, USA; 3 Department of Urology, Johns Hopkins University School of Medicine, Baltimore, MD 21231, USA

**Keywords:** COVID-19, lungs, acute respiratory distress syndrome, ground glass opacity, inflammation

## Abstract

**Simple Summary:**

According to the coronavirus virus resource center of Johns Hopkins Medicine, more than 75 million people are presently affected worldwide, including 1.7 million deaths due to severe acute respiratory syndrome since December 2019. Apart from the common symptoms similar to the common flu, a characteristic computed tomography (CT) feature i.e., Ground Glass Opacity (GGO) is highlighted in this article. GGOs have been observed in COVID-19 patients with severe symptoms including pneumonia in both lungs. It is important to reflect that GGO may indicate the onset of lung fibrosis and may be an indicative feature of high-risk subjects for developing lung cancer. In this article, the causes of the appearance of GGOs and their effects are mainly discussed, along with the brief immunopathogenesis of COVID-19 in comparison with other oncogenic viruses. In this pandemic situation, it is also important to consider the long-term effects of coronavirus infection and the ways to follow-up the patients who recovered from this disease.

**Abstract:**

Currently, the healthcare management systems are shattered throughout the world, even in the developed nations due to the COVID-19 viral outbreak. A substantial number of patients infected with SARS-CoV2 develop acute respiratory distress syndrome (ARDS) and need advanced healthcare facilities, including invasive mechanical ventilation. Intracellular infiltration of the SARS-CoV2 virus particles into the epithelial cells in lungs are facilitated by the spike glycoprotein (S Protein) on the outer side of the virus envelope, a membrane protein ACE2 (angiotensin-converting enzyme 2) and two proteases (TMPRSS2 and Furin) in the host cell. This virus has unprecedented effects on the immune system and induces a sudden upregulation of the levels of different pro-inflammatory cytokines. This can be a cause for the onset of pulmonary fibrosis in the lungs. Existence of a high concentration of inflammatory cytokines and viral load can also lead to numerous pathophysiological conditions. Although it is well established that cancer patients are among the high-risk population due to COVID-19-associated mortality, it is still unknown whether survivors of COVID-19-infected subjects are at high-risk population for developing cancer and whether any biologic and clinical features exist in post-COVID-19 individuals that might be related to carcinogenesis.

## 1. Introduction

The outbreak of COVID-19 infection was first reported in a Chinese city (Wuhan, Hubei province) and since then, this viral infection has been spreading throughout the world [1] The symptoms of COVID-19 infection were related to the previously known SARS (Severe Acute Respiratory Syndrome), MERS (Middle East Respiratory Syndrome), and influenza infections, which present with fever, sore throat, nasal congestion, body ache, and dry cough. Some of the COVID-19 patients also develop severe clinical disorders such as breathlessness, chest pain, and diarrhea. As of November 2020, United States has reported the greatest number of infected patients followed by India, Brazil, Russia, and many others with over 75 million infected individuals and more than 1.7 million deaths. According to the reports of World Health Organization (WHO), Centers for Disease Control and Prevention (CDC), Johns Hopkins, Mayo Clinic, and other medical institutions and organizations, older adults, males, and the patients with comorbid conditions such as cardiovascular disorders, diabetes, chronic respiratory disease, and cancer are more likely to have life-threatening conditions including pneumonia in both lungs, multi-organ failure, and various neurological disorders than others.

**Figure 1:**
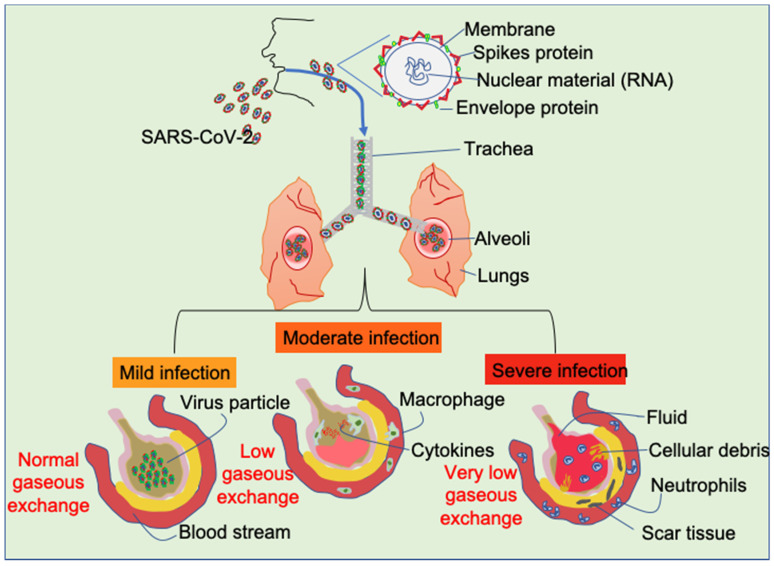
Effect of SARS-CoV-2 infection on the alveolar cells depending the severity of the infection.

SARS-CoV2 is a member of the *coronaviridae* family virus and has been reported to be associated with numerous coronavirus-associated diseases [2]. It is a single stranded RNA virus with a 5′ cap and 3′ poly A tail (30–32 kb). According to the National Cancer Institute (NCI), this virus can affect almost all organs and, most importantly, it causes acute respiratory disorder, as depicted in Figure 1. It is well established that numerous viral infections are causative to carcinogenesis [3] and previous reports suggest that viral infection-associated inflammation can initiate cellular transformation through activating/modulating several oncogenic signal transduction pathways [4]. Therefore, the association between COVID-19 survivals and cancer incidence in different organs may be a major point of concern to the cancer research community. Although no clinical and experimental data yet available, we speculate that SARS-CoV2 infection may activate some oncogenic pathways that may remain active even after the infection subsided.

A group of scientists performed complete genome sequencing of a novel coronavirus strain (SARS-CoV2) in the first week of January 2020. Since then, 1000 more sequences (complete and partial) have been submitted to the NCBI database by different scientists from several countries [5,6]. The viral genome typically consists of 6 open reading frames (ORFs). Although very few functional data available of these ORFs, ORF1a and ORF1b are comparatively more virulent [7]. These two ORFs transcribe and ultimately produce two polypeptides, pp1a and pp1b, respectively. Subsequently, these polypeptides are further processed by viral proteases to form 16 non-structural proteins (NSPs) [7,8]. The remaining ORFs are found to regulate the synthesis of different structural proteins of the viral capsid, including the spike, envelop, accessory protein chains etc. [9]. The structural proteins of the virus are very critical for the pathogenesis of the virus, and the NSPs are also known to blocking the host immune response [8].

In this review article, we have focused on the oncogenic viruses, the effect of COVID-19 on the immune system, ground glass opacity (GGO) and the potential neoplastic changes in the lungs due to SARS-CoV2 infection. Since the lungs are one of the most affected organs, clinicians generally evaluate infected patients’ lungs by computed tomography (CT). It was reported that chest CT findings of COVID-19 patients include the presence of the characteristic radiologic feature of GGO [10,11]. Presence of pulmonary GGO in CT image makes hazy opacity with not obscuring bronchial and vascular region, implying partial collapse of alveoli, partial filling of air space, and interstitial thickening. Previous reports have also suggested that CT findings of GGO were observed in high-risk populations of developing cancer, such as smokers [11,12].

## 2. Cancer-Related Viruses

The viruses with oncogenic potential have started to emerge in 1964 with the discovery of viral particles in Burkitt’s lymphomas by scientists Epstein and Barr [13]. According to a report of the WHO, approximately 10% of new cancer cases are related to viral infections in the year of 2012 in the world [14]. Viruses are of two types (DNA and RNA viruses) and generally cancer-causing viruses are called oncovirus. A list of well-known oncoviruses with their characteristic features and associated cancers are summarized in Table 1. Human papilloma virus (HPV), Hepatitis B virus (HBV), and Hepatitis C virus (HCV) cause the highest number of cancer incidence among the other oncoviruses worldwide, being linked to cervical/head & neck and hepatocellular cancer respectively. The other oncoviruses are namely Epstein–Barr virus (EBV), Human herpesvirus type 8 (KSHV), Human T-cell lymphotropic virus (HTLV-I), and Merkel cell polyomavirus (MCPyV) [15]. Generally, the immune system can eradicate most of the viruses after infection. However, in a subgroup of population, the virus can persist for a long time in the host tissue and can induce chronic inflammation and/or cellular transformation as a prerequisite for the development of certain cancers. For example, 80% of HPV-infected individuals are able to clear the virus within two years via the delayed adaptive immune response while 20% of infected people are not able to clear the infection that eventually, may lead to the development of cervical carcinoma [16].

HPVs drive carcinogenesis via E6, E7 proteins, and in addition to these proteins, some HPV sequences also encode a long control region (LCR) that facilitates the viral genome to integrate into the host genome. High risk HPVs have E7 proteins that degrade pRB proteins and facilitate retention of viral genes constantly at the S phase in the host cell cycle. While the host cell increases the p53 tumor suppressor protein, E6 proteins of HPV intervene proteasomal degradation of p53. These sequential events lead to dysregulation of the cell cycle, and the virus becomes immortal in squamous epithelial tissues of specific parts of the body like cervix, head and neck, etc.

HBV and HCV have a potential rendering tumor development via chronic inflammatory state when the immune system fails to clear the infection. Recurrent infection of the liver cells by the viruses leads to sustained reactive oxygen species (ROS) and reactive nitrogen species (RNS) production with possible genetic and epigenetic alterations finally resulting in tumorigenesis [17]. Specifically, the X protein driving oncogenesis was found in HBV, which utilizes Ras-Raf-MAPK, p53 pathways to promote tumorigenesis [18]. The occurrence of fibrosis, which is a result of extensive and repeated inflammation, stands for one of the main driving mechanisms in HBV- and HCV-related hepatocellular carcinoma (HCC) [19].

EBV and KSHV can cause lytic or latent infection and drive oncogenic transformation in the infected cells. EBV infection predominantly occurs in B cells with the expression of its partial integrated genome. EBV produces specific proteins like Epstein–Barr nuclear antigens 1 (EBNAs) and latent membrane protein 1–2 (LMP1-2), that activate some signaling pathways including JAK/STAT, MAPK, and NF-kb with eventually driving proliferation and sustained growth. KSHV has a broad tropism for B cells, endothelial cells, monocytes, and epithelial cells. Its oncogenic protein, namely latency-associated nuclear antigen (LANA) can drive malignant transformation by inactivation of tumor suppressors (such as p53 and RB) and increasing β-catenin level resulting in continuous cell cycle progression and proliferation in the host [20,21]. Similarly, Merkel cell polyomavirus (MCPyV)-encoded large T and small T antigens can inactivate RB and p53. Sustained production of the T antigens by MCPyV is required for the development of Merkel cell carcinoma (MCC), which is a type of skin cancer. Binding of Rb protein with large T antigen is needed for continuous growth of MCC and small T antigen is also involved in cellular malignant transformation [22]. Lastly, human T-cell leukemia-lymphoma virus-1 (HTLV-1) causing adult T cell leukemia/lymphoma (ATL) encodes Tax and HBZ proteins that induce dysregulated T cell proliferation. In early phase of the infection, Tax protein preserves T cell survival and induces strong cytotoxic T cell immune response. Over time, Tax protein is silenced, resulting in immune escape providing long latency period. HBZ protein, which was found constitutively expressed in ATL patients that eventually facilitates cellular transformation. Thus, ATL is driven by the expression of Tax, HBZ proteins, and somatic mutations resulting in inactivation of p53 function, deletion/ promoter hypermethylation of CDKN2A genes and other genetic alterations [23].

## 3. Infection of Lungs by SARS-CoV2

**Figure 2:**
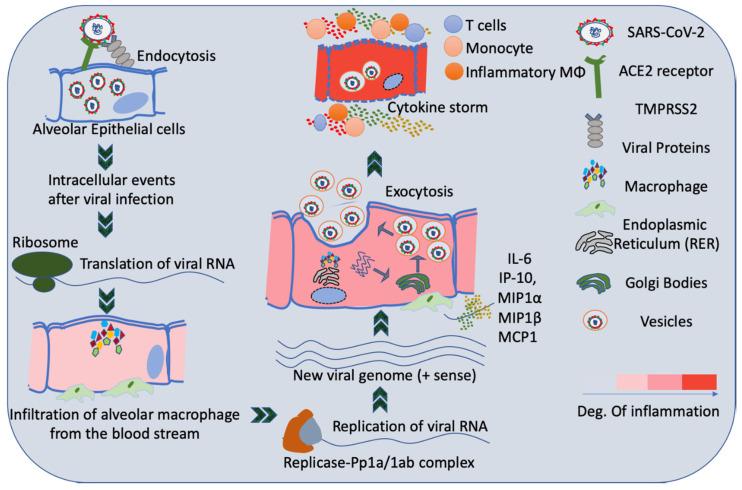
Schematic representation of sequential intracellular events in alveolar epithelial cells due to SARS-CoV-2 infection.

It has been reported that the viruses of the coronavirus family including SARS-CoV2 mainly enter the human body through the mucosa of nose and oropharynx, and some eventually get deposited in the lungs. Other organs expressing angiotensin-converting enzyme 2 (ACE2) receptors on the surface of the cells such as heart, kidney, and the intestines are also prone to be infected by the SARS-CoV2 [24]. ACE2 receptors, primarily a membrane-bound aminopeptidase, are the most probable binding region of the SARS-CoV2 particles to the host cell. The immature or undifferentiated cells have a very low level of expression of ACE2 compared to the matured or differentiated cells, which makes the matured cells more susceptible to the virus (Figure 2) [25,26].

## 4. Host Immune System and SARS-CoV2 Infection

SARS-CoV2 infection can induce both innate and adaptive immune response and allow excessive infiltration of several immune cells such as macrophages and T cells. This, in turn, results in the release of an excessive amount of different pro-inflammatory cytokines by these cells [27]. Accumulated clinical data suggest that SARS-CoV2 infection induces a sudden cytokine-burst in host immune system by activating the leukocytes through upregulating IL-6 and thus can affect different tissues of the body [28]. Different research reports suggest that acute respiratory disorder accompanied by the elevated concentration of several interleukins including IL-1β and IL-6 are present in the blood of the infected patients [29,30]. Pre-clinical reports from our group at Johns Hopkins Medicine and others also showed that deregulation of IL-6 can occur during some severe pathological instances like autoimmune disorders, cardiovascular disorders, and different types of cancer [31,32,33,34]. Clinical reports by Ridker et al. suggested that the baseline concentration of IL-6 in the plasma is found to be comparatively higher in patients with lung cancer than the control subjects [35]. By the mechanism of cytokine release syndrome (CRS), SARS-CoV2 infection may lead to the induction of severe symptoms associated with pneumonia and even multiple organ dysfunction [36]. Recent reports also suggest that SARS-CoV2 infection is also associated with rapid exhaustion of the NK cells and cytotoxic T cells [37] that may lead to rapid progression of the viral infection in the host, destruction of the immune system, and make the host susceptible to the secondary microbial infection. Therefore, survivors of COVID-19 disease may be more prone to several autoimmune disorders, COPD and cancer [38].

## 5. COVID-19 and Cancer

The immune system has been reported to be one of the most affected systems in the human by SARS-CoV2 infection [28], and a deregulated immune response may facilitate cancer initiation and progression [37]. A study from China indicates that cancer patients have nearly a four times higher risk of being infected by SARS-CoV2 compared to healthy individuals, and the fatality rate is about 30% for cancer patients compared to 3–4% for other subjects [37,39]. A study conducted at Memorial Sloan Kettering Cancer Center (MSKCC) reported that among 102 lung cancer patients having COVID-19, 62 patients developed severe symptoms, and 25 patients died with aggravated respiratory disorders [40]. Similar studies have also confirmed that lung cancer patients are at high risk in developing severe symptoms due to SARS-CoV2 infection and simultaneous ineffectiveness of the chemotherapeutic regime [41,42]. Individuals suffering from lung cancer generally have impaired alveolar function with upregulated expression of ACE2 receptor, immunosuppressive cytokines, and impaired function of cytotoxic immune cells [43,44]. Different health regulatory institutions including CDC, American Society of Clinical Oncology (ASCO) and American Association of Cancer Research (AACR) have suggested guidelines including safety measures to handle cancer patients suffering from SARS-CoV2 infection.

Apart from the concern about the existing cancer patients, previous research outcomes on the SARS and MERS patients raise a concern for a potential high-risk group of developing numerous health issues in severely affected patients by SARS-CoV2. As for example, it was reported that MERS can significantly upregulate different pro-oncogenic factors such as MEKK, MAPK, ATF2, C-Fos, p90RSK, Raf-1, and JNK mediated activation of AP-1 with simultaneous suppression of autophagy regulatory Bcl-2 family proteins [37,45]. Induction of endoplasmic reticulum (ER) stress was also found to be associated with the incidence of human coronavirus infection which may also lead to pro-oncogenic unfolded protein response in individuals with persistent infection and impaired autophagic signaling accompanied by weak immune response [46]. Therefore, in the next paragraphs, we have described some clinical findings that are evident in COVID-19 patients and these are also reported to be high-risk findings associated with lung cancer development.

## 6. Ground Glass Opacity (GGO) of Lung Determined by CT in COVID-19 Patients

Numerous studies have suggested that CT examination can be an easy and effective complementary tool in early detection of COVID-19 and distinguishing from other viral pneumonias [47]. Studies have shown a higher frequency of multifocal, bilateral, peripheral, and nonspecific distribution of GGO with sub-segmental patchy consolidations in SARS-CoV2-infected individuals compared to controls [48]. Another study of 22 SARS-CoV2-infected children revealed the significant abnormalities of the lungs detected by CT including GGO and consolidated areas in more than 50% of the patients [49].

GGO is a well-established term in a clinician’s handbook. It is usually observed in severe or persistent inflammation in lungs, including the manifestation of different abnormalities like interstitial fibrosis, pneumonia, granulomatosis, bronchiolitis and endometriosis [50,51]. The GGO nodules are of two types; the pure GGO that does not contain any solid material and part-solid GGO with solid component that is also considered as mixed GGO nodules [52]. It can be detected by CT as nodules accompanied with the abnormal opacity of bronchial structure that indicates the thickening of the alveolar interstitium with abnormal morphology of the alveolar walls, including the presence of fluid in the alveolar spaces [53]. It is still one of the most debatable topics among the multidisciplinary thoracic oncology team regarding the development of frank lung cancer originating from GGO. Migliore et al. reported that the occurrence of lung cancer in subjects with GGO is approximately 63%, that also indicated the biologic behavior of GGO is heterogeneous [54]. Although there is an immediate need to set uniform guidelines regarding the CT images, researchers and clinicians have agreed upon the fact that the presence of GGO for a long time in the lungs indicates a possibility of neoplastic development, and when the solid variants of GGOs are detected, the probability increases [54,55].

As noted above, GGOs (both pure and mixed GGO nodules) in the lungs can be correlated with the onset of lung cancer. The pure GGO nodules are related to adenocarcinoma in situ (AIS) and the mixed nodules are characterized as minimally invasive malignancy [56]. It was reported that the invasive nature of the malignant outgrowth depends on the ratio between the presence of pure and mixed GGO nodules [57]. By analyzing the CT images and the biopsy materials, clinicians can infer the nature of the GGO lesions. Such as lepidic pattern in the biopsy and presence of pure GGOs can potentially lead to AIS. However, it requires analysis of the clinical bio-specimen for final conclusion [56]. Apart from the correlation between the lung cancer subtypes and GGO, reports also suggest that there can be potential relationship between the mutation profile of some key oncogenic drivers (such as EGFR and KRAS) and the occurrence of specific GGO subtype. A recent study determined that 2/3rd of the patients who exhibited pure GGO characteristics have amplified expression of EGFR [58]. Presence of the pure GGOs in the CT reports is less common for patients having KRAS mutation and/or deregulated expression of ALK [59]. More controlled studies are required to conclude regarding the correlation between cancer subtype or mutational behavior and presence of GGO subtype. Overall, it can be inferred that in the early detection of lung adenocarcinoma (LUAD), the presence of GGO nodules plays a critical role in decision making for the clinicians. CT findings from different patients also suggest that GGO nodules can also be observed in patients with benign tumors and in patients having adenomatous hyperplasia lesions. We recently investigated the likelihood of detecting aberrant DNA promoter methylation of a panel of TSGs (DCC, Kif1a, NISCH, and RAR-β) in plasma samples of patients with abnormalities of the lung detected upon CT scan [51]. Twenty-two percent of patients with GGO exhibited methylation of at least one gene. Our study concluded that methylation biomarkers studies can distinguish between cancerous and noncancerous in abnormal CT findings. In a separate study [60], we perform targeted next-generation sequencing on multifocal atypical adenomatous hyperplasia (AAH) and different zones of histologic progression within AISs and minimally invasive adenocarcinoma (MIA) in the lungs. Multi-region sequencing demonstrated different genetic drivers within the same tumor and reveals that clonal expansion is an early event of tumorigenesis. We find that KRAS, TP53, and EGFR mutations are indicators of malignant transition. Therefore, GGOs are considered premalignant lesions and our analysis identified substantial molecular alterations in these early lesions that may drive the carcinogenesis process over time.

The widespread GGO findings in CT images of COVID-19 patients have raised the probability of developing lung cancer and need to follow up at regular interval for early detection of any kind of pre-neoplastic lesions [61]. In addition to imaging modalities, several investigators have attempted to identify liquid biopsy-based biomarkers for the detection of these early lesions. Interestingly, we are able to detect a panel of epigenetic and genetic biomarkers in the plasma of subjects who were diagnosed with these early lesions [51,60]. Other investigators also reported similar findings [62,63]. In the future, selected COVID-19 survivors need to be followed up for early detection favorably by low-cost non-invasive approaches such as measuring genetic and epigenetic biomarkers in blood and/or broncho-alveolar lavage.

## 7. Involvement of Inflammatory Cascades in Carcinogenesis and COVID-19 Disease

Epidemiologic and clinical studies have shown the relationship between chronic infection/inflammation and cancer that usually follow multi-step process [64]. Notably, association of cancer and inflammation is a highly dynamic process in which chronic inflammation initially can lead to neoplastic changes and subsequently, these oncogenic changes in cells provide inflammation rich tumor microenvironment [65,66]. Around 25% of cancers are referred to have a keen relationship with chronic inflammation caused by infection, autoimmune diseases, and irritants [67]. Well-known examples attributed to cancer-related inflammation and subsequently lead to cancers are *H. pylori*-induced gastric inflammation-stomach cancer, chronic hepatitis-liver cancer, chronic pancreatitis-pancreas cancer, cholecystitis-gallbladder cancer, chronic prostatitis-prostate cancer, inflammatory bowel disease-colorectal cancer, and inflammation/fibrosis via infection, irritants, and smoking-induced lung cancer [67].

Various cells of the immune system are recruited to the site of infection upon SARS-CoV2 viral entry, which include lymphocytes, monocytes, macrophages, neutrophils, mast cells, eosinophils, dendritic cells, and natural killer cells etc. Pro-inflammatory cytokines are the frontline effector molecules in any kind of microbial infection and SARS-CoV2 infection has no exception as it was mentioned earlier. In the pathogenesis of SARS-CoV2 infection, the elevated expression of different cytokines and chemokines (IL-6, IL-1α, IL-1β, IL-2, IL-8, IL-10, IL-17, IFNγ, TNFα, IP-10, M-CSF, G-CSF, MCP-1, MIPs, VEGF) play a critical role in the antiviral response against SARS-CoV2 and however, also dysregulation of immune activity [68]. Specifically, in the blood of COVID-19-infected patients, IL-6, IFNγ, MCP1, and IP-10 were found elevated [69]. In another study [70], IL-2, IL-7, IL-10, IP-10, MCP1, MIP1A, G-SCF, and TNFα showed higher plasma levels in severe COVID-19 patients. Sharp and dramatic increase in circulating levels of these pro-inflammatory cytokines can be defined as a cytokine storm, which is mainly through IL-6 in COVID-19 patients.

Pro-inflammatory cytokines are also involved in several mechanisms of inflammation-associated cancer. These mechanisms are via inducing DNA damage, excessive production of angiogenic molecules (VEGF, IL-8, NO), ICAM-1 and VCAM-1, and stimulation of cell proliferation and inhibition of apoptosis [17,64,67]. Immune cells can produce reactive oxygen species (ROS) and reactive nitrogen species (RNS). Productions of ROS and RNS may drive inflammation-induced carcinogenesis by rendering DNA damage, alterations in cellular proteins (such as inactivation of Rb protein in colorectal cancer) and lipids, and subsequently activation of oncogenes and/or repressing TSGs [67]. For example, 8-oxo-dG and 8-NG, which are metabolites of ROS and RNS causing DNA damage in cells, have been shown in the initiation of inflammation-driven carcinogenesis [71,72]. The 8-NG product was also reported to be formed via iNOS production in patient samples at infection/inflammation site of *H. pylori*, *HBV*, *HCV*, *HPV*, *EBV*, and *Schistosoma haematobium* (SH), *Opisthorchis viverrini* (OV) [72]. When abundant free DNA coming from died cells in infection site confronts with a high content of oxygen in the lung vessels reveals more 8-oxo-dG, which may increase the risk of cellular transformation in the lungs [73].

Infection/inflammation can cause molecular changes and recruitment of inflammatory cytokines/chemokines in lung tissue microenvironment. Cytokines affect cells via activation of transcription factors like STATs, NF-kB, and AP-1 by binding their specific receptors and subsequent activation of intracellular kinases like Janus activated kinase (JAK), phosphatidylinositol-3-kinase (PI3K)/Akt, and MAP kinases [74]. TNFα is one of the major pro-inflammatory cytokines presented in COVID-19 patients, which has paradoxical effects on tumor growth. It was reported that intra-tumoral levels of TNF are likely insufficient to regress tumor [75] and chronic exposure to TNFα increases the risk of tumor development and progression. Along with that, significantly increased levels of TNFα was found in the pre-neoplastic lesions of several cancers [76,77]. TNFα also has a potential to render DNA damage and cellular transformation through ROS in inflammation-associated carcinogenesis [78].

The increased plasma level of IL-6 and IL-1β in COVID-19 patients and the long-term effect of short period of elevated expression of these cytokines not yet completely known. IL-6 is an important orchestrator for inflammation, it promotes several pro-oncogenic pathways and evasion from immunity, and suppresses the host antitumor immune responses [79]. IL-6 maintains the expression of gene levels for the cell cycle progression and regressing of apoptosis. Specifically, IL-6 regulates AKT mediated cell survival pathways [80]. Increased levels of IL-6 and IL-1β was also found in inflammation-related gastric cancer lesions compared to normal mucosal tissue [81]. On the other hand, IL-6, produced by cancer-associated fibroblasts (CAFs), induces invasion, metastasis, and EMT properties in lung cancer via STAT3 pathway [82]. Similarly, IL-6 expressed by tumor-associated macrophages (TAMs) in KRAS driven lung cancer model causes activation of STAT3 pathway with tumor progression [83]. Interestingly, in another study, alveolar macrophages were found responsible for the progression of EGFR mutant LUAD [84].

Other cytokines such as IL-1α, IL-1β, and IL-17 that are also known to be elevated due to COVID-19 disease may involve the regulation of inflammation-related carcinogenesis. While IL-1α is related to more aggressive tumor formation with increased cell proliferation and angiogenesis [85], IL-1β has an important role via proliferating epithelial cells in lung carcinogenesis. Also, IL-1β was found inversely correlated with progression-free survival in advanced NSCLC patients [86]. IL-17 is proved to actively promote angiogenesis and tumor growth in human NSCLC in vivo [87]. Therefore, it is possible that COVID-19 patient may have an increased chance to have such a cellular change over time.

The intricate mechanism behind the development of life threatening acute respiratory disorders is still being elucidated. However, early studies have shown the interplay of the CD4^+^ T cell-related immune response making responsible for the damage of the lungs in COVID-19 patients [88]. An immunogenic pathway involving the activation of CCL2- and CXCR2-associated inflammatory response can cause long term damage in the lungs, including pulmonary fibrosis [89,90]. The pathway driven by the CCL2/CXCR2 axis activates the expression of TNFα, iNOS, and deregulate the oxidative homeostasis in the lungs leading to progressive tissue damage [91]. The CCL2/CXCR2 axis is also a well-studied oncogenic pathway that regulates the immune response in the tumor microenvironment and affects the progression of resistant and metastatic tumor [92]. This has led the scientific community to consider the onset of malignant transformation as a long-term side effect of COVID-19 [93]. From the early days of the disease identification and characterization, it was noted by the clinicians that pulmonary fibrosis is one of the major damages caused due to the coronavirus infection [94,95]. Reports suggest that the onset of pulmonary fibrosis in COVID-19 patients is primarily due to direct alveolar damage and the cytokine storm, and it can be regarded as the consequence of acute respiratory distress syndrome (ARDS] [96,97]. The effect of pulmonary fibrosis or severe damage in the lung can also be observed in later times in the absence of the infection, and this long-term effect can be attributed to the persistent hyper-inflammatory state [69,98]. Older individuals are at higher risk of developing such symptoms after severe SARS-CoV2 infection, but reports are also noticed this in relatively young and physically fit individuals without having any underlying conditions [93,99].

In a recent study from Shanghai Medical College, China reported the effect of chemotherapy in 55 LUAD patients that were previously shown to have GGO features. They concluded that these patients are non-responsive to chemotherapy. Therefore, it can be speculated that the LUAD patients who already have had GGO like pathological features are at high risk of that their regular therapeutic regime may not be effective due to additional pathological condition [61]. Cancer patients treated with chemotherapies have compromised immune system that makes this group of patients more vulnerable to SARS-CoV2 infection. Other patients with compromised health conditions such as patients with heart disease, asthma, diabetes, liver disease, and renal disease are at high-risk for mortality due to the infection. Recently Lung Cancer Foundation (USA) reported that patients with lung cancer have shown worse outcome due to COVID-19 disease and considerably higher mortality rate compared to other groups of patients with previously reported deleterious conditions [100]. Additionally, lung cancer patients who are already being treated with different immune checkpoint blockers are at potentially high risk in developing pneumonitis, which is similar to the onset of pneumonia due to the infection as revealed by the CT findings [43]. In addition to the therapeutic perspective, because of overlap in CT feature between the manifestation COVID-19 and lung cancer progression, it will be a challenge for the clinicians to monitor the progression of both the diseases and choose the therapeutic approach [101,102,103].

Other important noteworthy aspects in relation to the cytokine storm in the COVID-19 patients are the onset of myalgia, anorexia and subsequent muscle wasting. Altogether, these symptoms clinically refer to the cachexia and sarcopenia are also frequently observed in patients with severe symptoms. Onset of cachexia leads to unfolded protein response (ER stress), including overproduction of several cytokines like TNFα, IL-1, and IL-6, and can activate different critical cancer-associated regulatory pathways by activating key signaling molecules like NF-κB, STAT3, IGF-1, AKT, mTOR and many others [104,105,106,107]. There are also reports like the involvement of cranial nerve in the severely affected COVID-19 patients causing oropharyngeal dysphagia as well as aspiration pneumonia [108], which contribute to muscle atrophy or cachexia. These possess extreme threats to patients with preexisting cancer as well as severe COVID-19 patients with no preexisting disorders. Continuous cytokine storm, proteolytic signaling mechanisms and onset of cachexia imprints a risk of premalignant transformation in the affected organ [109,110].

## 8. Summary and Future Perspectives

The world is now busy to manage the present situation of COVID-19 outbreak. However, it is necessary to make plans to manage different health conditions for the post-COVID-19 outbreak time as COVID-19 survivors may develop numerous chronic health hazards including cancer, specifically lung cancer. The onset of pulmonary fibrosis due to COVID-19 infection leads us to speculate one of the worst outcomes of COVID-19 survivors. Induction of cytokine storm may facilitate pulmonary fibrosis development that may reflect the appearance of GGO in the lungs of COVID-19 patients [111,112,113]. The CT findings of GGO may also be correlated with increased expression of ACE2 and need to be analyzed in future studies. In the lungs of COVID-19 patients, different stages of pulmonary interstitial damages along with parenchymal changes are observed along with the appearance of GGO in CT images [10]. Among other CT manifestations of lung, the most commonly appeared features in the COVID-19 patients are consolidation, reticular pattern, crazy paving pattern, air bronchogram including the airway changes, pleural changes, sub-pleural curvilinear line, and fibrosis [114]. These CT manifestations are quite similar that are also observed in pre-neoplastic lesions of LUAD. Earlier reports on human samples and experimental animals suggested that many survivors of SARS-CoV infection leads to overexpression of EGFR and occurrence of pulmonary fibrosis [115]. As noted previously, we identified that KRAS, TP53, and EGFR mutations occurred in pre-neoplastic lesions of LUAD [60]. Another recent study reported the involvement of CD147 in the lungs of SARS-CoV-2-infected patients [116]. Expression of CD147 was reported to be related to tumorigenesis process through the activation/modulation of the activity of MMPs and ABC drug transporters [117]. Additionally, enhanced expression of CD147 is also associated with the increase in cellular stemness (plasticity). Future studies will explore the molecular alterations associated with SARS-CoV-2 infections in the lungs, and that will allow us to understand whether this infection is associated with molecular alterations leading to the initiation of lung cancer development.

SARS-CoV2 was reported as a cytopathic virus [118] that induces host cell lysis following infection. As commonly seen in cytopathic viruses, SARS-CoV2 causes pyroptosis, which is a programmed host cell death with high and rapid immunologic response and is considered to have a central role in cytokine storm [113,119]. This pyroptosis is one of the possible onsets of the virus pathogenesis that results in increased IL-1β level in the serum of COVID-19 patients [120]. In recent years, cytopathic viruses have been considered for causing not only cell lysis but also surviving host cells with/without injury. Additionally, for a given virus, it is not known whether additional host cell fates occur due to exposure of different viral properties caused by intrinsic pathogenesis such as mutations or uncompleted viral packaging [121]. It is proposed that possible other cell fates consist of important potential changes for the behavior of the host cells to the virus such as transmissibility, immune response, signaling pathways, and viral fitness [122]. These other cell fates might render contribution to excessive inflammation, altered response to secondary infection, serving long term source of viral proteins and manipulating tissue structure and functions [121]. Interestingly, a recent clinical study showed that SARS-CoV2 viral elements and inclusion structures were found in endothelial cells through vascular bed of lung, kidney, liver, heart, and small intestine, referring to direct evidence of endothelium inflammation; endothelitis [123]. This result also supports the importance of binding of SARS-CoV2 to ACE2 receptors of the host cells and determine possible infection sites in the body. Theoretically, SARS-CoV2 is potentially able to infect ACE2 receptor expressed cells wherever it is. Since endothelium is the lining of all blood vessels of the body, COVID-19 may also increase the risk of some other complications by disrupting endothelial function, such as serious inflammation with associated tissue edema, disseminated intravascular coagulation, coagulopathies, and acute organ failures [123]. If COVID-19 disease causes endothelial dysfunction for a long time period that has not been known yet, endothelial dysfunction-related diseases should be taken into account in the future, including cardiovascular diseases and cancer.

As of now, over 75 million COVID-19 cases have been detected in developed and developing countries, and these numbers may be much more if the number of tests can be increased throughout the world. In regard to surveillance of different diseases including cancer in COVID-19 survivors, the countries that have established screening programs should be aware of these possibilities and take action for screening of different pathological conditions after the acute infection. The COVID-19 pandemic has been in our life since December 2019. Even though SARS-CoV2, as the pathogen of COVID-19 disease, has not been reported as a causative virus for tumorigenesis, it is also important to find out co-infection with other cancer-related pathogens and/or additional co-factors (such as carcinogens) may facilitate pre-cancerous lesions in the setting of infected host cells and tissues in the long time courses. Although it is not likely to predict that all GGO nodules in the lung due to the infection will persist for a long time, there are several reports providing follow-up CT results (mainly GGO) warning long term sequelae in COVID-19 patients [55,124,125]. GGO nodules, if persisting in COVID-19 patients, might have potential to transform into lung cancer in coexistence with specific conditions such as smoking, certain genetic susceptibility, and other risk factors.

## Figures and Tables

**Table 1:** List of human viruses that possess potential threats for malignant transformation.

Virus	Nuclear Material	Route of Infection	Risk of Cancer
Epstein-Barr virus (EBV)	DNA Virus	Respiratory secretions, Saliva	Lymphoma, nasopharynx, gastric cancer
Hepatitis B virus (HBV)		Blood and sexual route, perinatal	Hepatocellular carcinoma
Human Papilloma virus (HPV)		Sexual route	Anal, cervical, head and neck, oral, penile, vaginal, vulvar
Human herpes virus 8 (HHV-8)		Saliva, sexual route, blood	Kaposi sarcoma
Merkel cell polyomavirus (MCV)		Respiratory secretions, saliva, skin?	Merkel cell carcinoma (MCC)
Hepatitis C virus (HCV)	RNA Virus	Blood and body fluids	Hepatocellular carcinoma
Human T-lymphotrophic virus-1 (HTLV-1)		semen, vaginal fluids, blood and breast milk	T-cell leukemia/lymphoma (ATL)
